# Slit–Robo signalling in heart development

**DOI:** 10.1093/cvr/cvy061

**Published:** 2018-03-10

**Authors:** Juanjuan Zhao, Mathilda T M Mommersteeg

**Affiliations:** Burdon Sanderson Cardiac Science Centre, Department of Physiology, Anatomy and Genetics, Burdon Sanderson Cardiac Science Centre, University of Oxford, South Parks Road, Oxford OX1 3PT, UK

**Keywords:** Congenital heart defects, Heart development, Slit–Robo signalling, Ventricular septal defects, Bicuspid aortic valves

## Abstract

The Slit ligands and their Robo receptors are well-known for their roles during axon guidance in the central nervous system but are still relatively unknown in the cardiac field. However, data from different animal models suggest a broad involvement of the pathway in many aspects of heart development, from cardiac cell migration and alignment, lumen formation, chamber formation, to the formation of the ventricular septum, semilunar and atrioventricular valves, caval veins, and pericardium. Absence of one or more of the genes in the pathway results in defects ranging from bicuspid aortic valves to ventricular septal defects and abnormal venous connections to the heart. Congenital heart defects are the most common congenital malformations found in life new-born babies and progress in methods for large scale human genetic testing has significantly enhanced the identification of new causative genes involved in human congenital heart disease. Recently, loss of function variants in *ROBO1* have also been linked to ventricular septal defects and tetralogy of Fallot in patients. Here, we will give an overview of the role of the Slit–Robo signalling pathway in *Drosophila*, zebrafish, and mouse heart development. The extent of these data warrant further attention on the SLIT–ROBO signalling pathway as a candidate for an array of human congenital heart defects.

## 1. Congenital heart disease and the role of the Slit–Robo signalling pathway

Congenital heart disease affects ∼1% of all live births^1^ and includes a wide range of conditions such as ventricular septal defects, atrial septal defects, bicuspid aortic valves, and tetralogy of Fallot.^1^^,^^2^ Advances in cardiac developmental biology have significantly improved our understanding of the signalling pathways and transcriptional networks underlying heart development. Additionally, improved human genetic testing on a large scale using SNP arrays and whole exome sequencing has opened up the possibility of searching for new causative genes, while targeted resequencing facilitates identifying SNPs in genes already well-known to be mutated in congenital heart disease.^3^^,^^4^ Recently, using whole exome sequencing, loss of function variants in *ROBO1* have been linked to ventricular septal defects and tetralogy of Fallot.^5^ Although these variants have not yet been tested for causal implications, the defects found in patients with *ROBO1* variants are very similar to those found in mouse models mutant for the *Slit* and *Robo* genes.^5^^,^^6^ Mouse models in particular suggest a thus far undetected involvement of the pathway in many more aspects of human congenital heart disease, from bicuspid aortic valves to abnormal venous connections to the heart.^6–8^ These data warrant further attention on the SLIT–ROBO signalling pathway as a candidate for an array of congenital heart defects. The Slit ligands and their Robo receptors are still relatively unknown in the cardiac field. However, the ligands and receptors of this pathway link to some of the most well-known genes mutated in congenital heart disease, including *TBX1* (DiGeorge syndrome), *CHD7* (CHARGE syndrome), *TBX5* (Holt–Oram syndrome), *NKX2.5*, and *NOTCH1*.^6^^,^^8–10^ Syndromes showing coexistence of all or some of the defects observed in the *Robo* and *Slit* mutant mice have been described, including bicuspid aortic valves, septal defects, anomalous inferior caval veins, partial absence of the pericardium, and diaphragmatic hernias^7^^,^^11–13^ but no causative genes have yet been identified.

Here, we highlight the extensive roles of the Slit–Robo pathway during heart development, from *Drosophila* to mouse models, showing its possible involvement in the development of a large range of congenital heart disease.

## 2. The Slit–Robo signalling pathway: structure and interactions of the ligands and receptors

Slit proteins are large secreted extracellular molecules, which were initially discovered in a genetic screen in *Drosophila melanogaster*^14^ and subsequently found to be midline axon repellents during the development of the central nervous system.^15^^,^^16^ During development, most axons cross over the midline to innervate the contralateral side of the body. Slit was found to be expressed at the midline and a mutation in the gene was shown to cause axons to enter but never to leave the midline, indicating Slit acts as a midline repellent.^16^ The receptor for Slit was identified as the single-pass transmembrane roundabout (Robo) receptor.^17^^,^^18^ The first *robo* gene was named after the phenotype of *Drosophila* mutants in which axons were observed to inappropriately cross and re-cross the midline, resembling the circular traffic junction (ROundaBOut). The growth cones of the axons that will cross the midline to the contralateral side initially do not express the Robo receptor and, therefore, are able to cross the Slit-expressing midline. As soon as the axons have crossed to the contralateral side, their growth cones start to express Robo, preventing them from re-crossing.^18^

**Figure 1:**
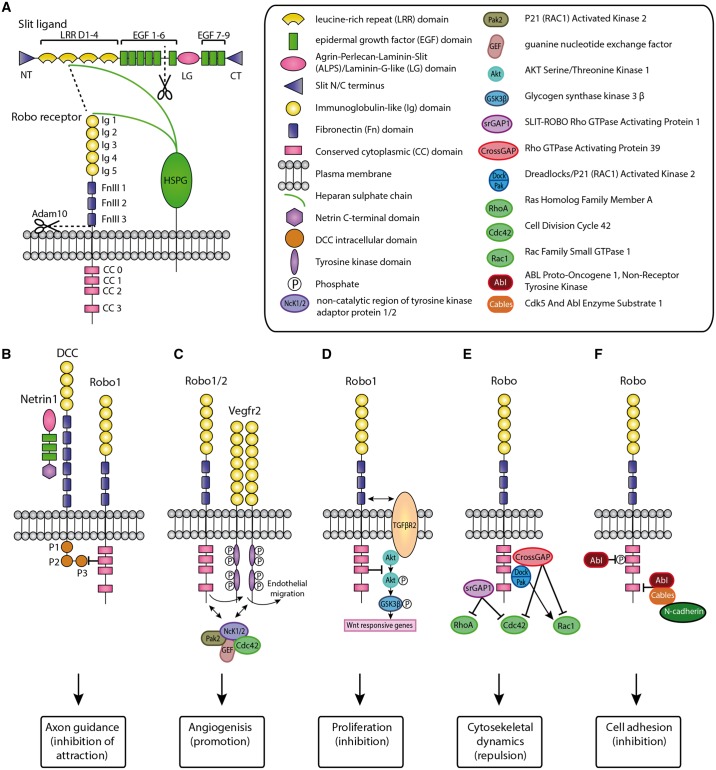
Schematic representation of the Slit ligands and Robo receptors.^19^^,^^21^^,^^22^^,^^32^^,^^35–44^*A*, Slit binds to the Robo Ig1 domain with their LRR D2 domain. The Robo4 receptor only has two immunoglobulin-like domains and two fibronectin repeats. Interaction between Slits and Robos can be stabilized by HSPGs. *B–E*, Examples of interactions between Robo and other membrane receptors, as well as downstream signalling casacades that are known to be involved in heart development or that have been identified in other organsystems and might also play a role during heart formation. *B*, The Robo1 intracellular domain interacts with the intracellular domain of the Dcc receptor, inhibiting Netrin-induced cellular attraction.^48^*C*, Robo1/2, together with Vegfr2, promote endothelial cell polarity during sprouting angiogenesis.^54^ Robo1 also promotes auto-phosphorylation of Vegfr2 to regulate endocardial migration.^53^*D*, Tgfβr2 interacts with Robo1 to elevate Robo1 expression, resulting in inhibition of transcription of Wnt-responsive genes.^52^*E*, Signalling through Robo can be propagated intracellular to GTPases of the Rho family such as RhoA, Rac1, and Cdc42, which are small guanosine-5′-triphosphate (GTP)-binding proteins that regulate cytoskeletal dynamics to control cell motion.^55^ The adaptor protein Dreadlocks (Dock) links Robo to the GTPases in both *Drosophila* and mammals.^56^^,^^57^*E*, Cytoplasmic kinase Abelson (Abl) phospholates the Robo CC1 domain to antagonize Robo activity.^65^ Additionally, Abl and its substrate cables link Robo to N-cadherin, which leads to the detachment of β-catenin from N-cadherin. Both inhibit cell adhesion.^92^

A single *slit* and three *robo* genes have been identified in *Drosophila*.^17^^,^^18^ In mammals, there are three *Slit* genes (*Slit1-3*), all of which are expressed in the nervous system as well as in a broad range of other organs.^19^^,^^20^ All Slits have a similar protein structure: four stretches of leucine-rich repeat (LRR) domains; seven to nine epidermal growth factor (EGF) repeats; an Agrin-Perlecan-Laminin-Slit/Laminin-G-like domain; and a C-terminal cysteine knot (*Figure 1A*).^21^ Slits are able to homodimerize through their fourth LRR domain,^22^ as well as able to bind to other extracellular matrix molecules, such as Netrin1,^23^ Glypican,^24^ Syndecan,^25^^,^^26^ Type IV Collagens,^27^ and Dystroglycan.^28^ Furthermore, Slits are able to bind to Dscam1 (down syndrome cell adhesion Molecule 1),^29^ and the Eva1C receptor.^30^^,^^31^ Slit2 protein can be cleaved into a long N-terminal and a short C-terminal fragment at the proteolytic site between the fifth and sixth EGF domain.^32^ The N-terminal fragment stays associated with the cell surface and, like full-length Slit, binds Robo to induce chemorepulsion,^32^ whereas the C-terminal fragment enters the extracellular space and binds the basement membrane scaffolding protein Dystroglycan^28^ and the Plexin A1 receptor.^33^ In most vertebrates, there are three Robo receptors Robo1 (Dutt1), Robo2, and Robo3 (Rig1).^21^ However, in zebrafish and mammals, a fourth Robo receptor was discovered, Robo4, also known as Magic Roundabout.^34^ The Robo receptors contain five immunoglobulin-like domains (two in Robo4), three fibronectin repeats (two in Robo4), a transmembrane domain as well as four conserved cytoplasmic domains (*Figure 1A*). The cytoplasmic domains do not have autocatalytic or enzymatic activity but interact with downstream signalling molecules.^35^ The receptors can be alternatively spliced,^36–38^ and undergo ectodomain shedding by Adam proteases, a process required for recruiting intracellular signalling molecules.^39–41^ Further cleavage by γ-secretase results in a C-terminal fragment that translocates to the nucleus, although function of this fragment is still unknown.^41^ Robos bind to the concave face of the Slit LRR2 domains through their Ig1 domain.^42^^,^^43^ The interaction between Slits and Robos can be stabilized by complex formation with heparan sulfate proteoglycans (HSPGs) (*Figure 1A*).^22^^,^^44^

In *Drosophila*, Robo was found to be able to bind directly, *in trans*, to Robo2 on neighbouring cells, inhibiting Slit-mediated repulsion.^45^ Whereas in mammals, Robo1 and Robo2 have been identified to function as (*cis*) homo and heterodimers.^46^^,^^47^ The Robo receptors have been shown to interact with a number of other membrane receptors, including Netrin receptor deleted in colorectal carcinoma (DCC)^48^ Sdf1 (Cxcl12) receptor Cxcr4,^49^ Neurexin IV,^50^ Neuropilin1,^51^ TgfbrII,^52^ Vegfr2,^53^^,^^54^ and Dscam.^47^ Upon binding of Slit ligand, signalling through the Robo receptor can be propagated intracellular to regulate axon guidance, cytoskeletal dynamics, cell adhesion, angiogenesis, and proliferation (*Figure 1B–F*).^55–57^

Although Slits and Robos were initially characterized as repulsive guidance cues for neuronal axons,^16^^,^^58^ further studies using different animal models have since found important roles for the pathway during many aspects of heart development, from lumen formation to valve development, which will be discussed in detail.

## 3. Slit–Robo signalling is required for heart cell migration, alignment, and lumen formation in *Drosophila*

**Figure 2:**
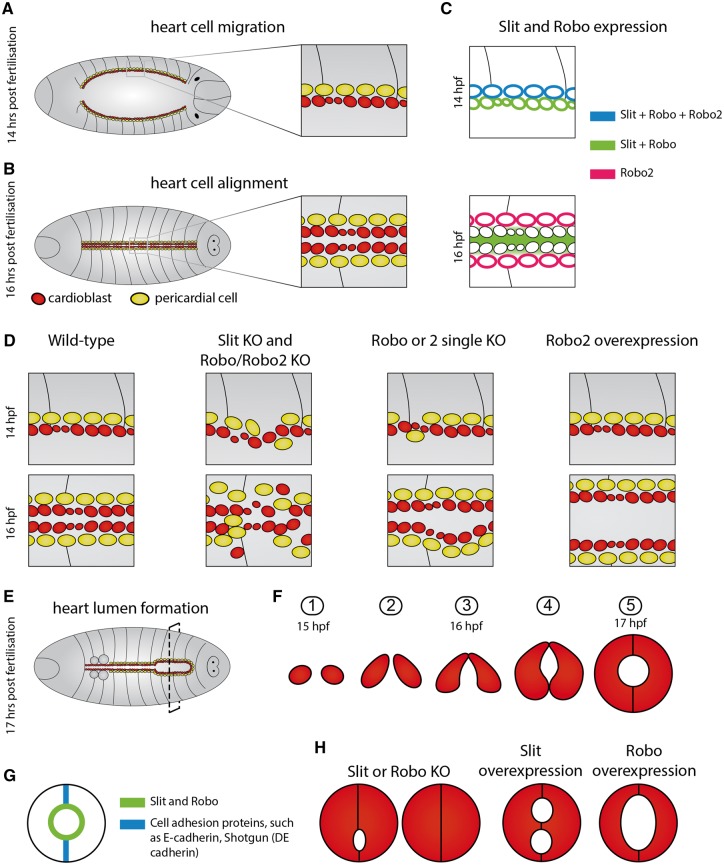
Schematic representation of the role of Slit–Robo signalling during *Drosophila* heart development based on^62–67^. *A*, 14 h post-fertilization *Drosophila*, during migration of the two rows of cardioblasts (red) and pericardial cells (yellow) to the midline. Main cardioblasts are depicted slightly larger than the ostia cardioblasts. *B*, 16 h post-fertilization *Drosophila*, during cardioblast and pericardial cell alignment at the midline. *C*, Expression of Slit, Robo, and Robo2 during these stages. *D*, The wild-type phenotype compared with the different mutant phenotypes during migration and alignment. *E*, 17 h post-fertilization *Drosophila*, during cardiac lumen formation. Dotted line indicates the line of sectioning to obtain the transversal view through the two contralateral cardioblasts as shown under Number 5. *F*, The process of lumen formation. The two cardioblasts first join on their dorsal sides, followed by the ventral side. The middle section of the membrane never meets, resulting in the lumen being formed. *G*, Slit and Robo expression becomes restricted to the luminal side, whereas cell adhesion molecules become localized where the cardioblasts join on their dorsal and ventral side. *H*, Phenotypes as observed in mutants for Slit or Robo.

The fruit fly, *D.melanogaster*, has an open circulatory system, with a simple linear tube-like heart that pumps the haemolymph from posterior in the body towards the anterior region.^59^ Although this anatomy is very different from the adult vertebrate heart, the tube-like heart resembles the initial heart tube of the developing vertebrate heart. Seventy-five percentage of all human disease genes have homologues in *Drosophila* and several genes causing human congenital heart disease have similar roles during *Drosophila* heart development.^59^^,^^60^ The cardiac progenitors originate from mesodermal cells that undergo a mesenchymal-to-epithelial transition and migrate towards the midline as two bilateral sheets of cells (*Figure 2A* and *B*). Both sheets are composed of an inner row of contractile cardioblasts and an outer row of pericardial cells.^61^ The two sheets meet each other at the midline, where the cardioblasts make adherens junctions and start forming a lumen that enlarges during the late stages of embryogenesis (*Figure 2E* and *F*).

The earliest expression of *slit* mRNA is observed in the lateral mesoderm.^62^ As soon as the cells are migrating towards the midline, both Slit and Robo (the homologue of vertebrate Robo1) protein are uniformly expressed on the cardioblasts.^63^^,^^64^ By the time the rows fuse, Slit protein has shifted location to uniquely localize on the apical side of the cardioblasts, where the ligand accumulates between the two rows of cells (*Figure 2C*).^62–64^ Robo now mainly concentrates on the apical surface of the cardioblasts, whereas robo2 seems to remain limited to the surface of pericardial cells,^63^^,^^64^ although cardioblast mRNA expression has been reported.^62^ Absence of *slit*, *robo2*, or myocardial overexpression of *robo2* results in delayed cardioblast and pericardial cell migration.^62^^,^^64^ Furthermore, absence of *slit*, both *robo* and *robo2*, or overexpression of *robo* in mesodermal cells causes the cardioblasts and pericardial cells to fail to align properly in the midline.^63^ The normally highly regular row of cardioblasts is already perturbed before they reach the midline, resulting in gaps between the cells and cardioblasts to become inappropriately interspersed within the pericardial cells (*Figure 2D*).^62–64^ Slit and Robo expression specifically within cardioblasts is sufficient to rescue the *slit* and *robo*/*robo2* knock out phenotype.^63^^,^^64^ These results indicate the importance of Slit–Robo signalling for heart cell migration and adhesion.

After the two rows of cardioblasts have aligned in the midline, lumen formation starts. Contralateral pairs of cardioblasts make specific dorsal and ventral contacts between their opposing apical sides to form the lumen. During this process, both Slit and Robo become localized on the part of the cardioblast membrane that will form the lumen (*Figure 2G*).^65^^,^^66^ During normal development, cell adhesion proteins, such as E-cadherin are specifically located at the dorsal and ventral side of the membrane of the cardioblast, exactly where the two contralateral cardioblasts make cell contact (*Figure 2G*), resulting in strong adhesion between the two cells.^65^ However, the presence of Slit and Robo on the medial part of the cell membrane and in between the cells, ensures that the cell membranes of the two contralateral cells are repulsed and a lumen is formed. Embryos overexpressing *slit* show ectopic lumen formation,^65^ whereas *robo* overexpression causes a larger lumen.^66^ In mutant embryos lacking either *slit* or both *robo* and *robo2*, the cardioblasts do not become triangular but remain rounded. As a result, the contralateral cells come into contact with each other along most of their apposing surfaces, blocking lumen formation (*Figure 2H*).^67^ These results indicate the important role of Slit–Robo mediated local repulsion in creating the lumen of the heart. Additionally, Slit–Robo signalling is important for outflow tract formation of the *Drosophila* heart. In *slit* and double *robo/robo2* mutant embryos, heart-anchoring cell (cells that share similarities to cardiac neural crest cells in vertebrates) migration is delayed or disrupted, and cardiac outflow tract muscles do not attach to the tip of the heart.^68^

## 4. Cardiac progenitor migration and lumen formation are also disturbed in *slit* or *robo* knock-down zebrafish

**Figure 3:**
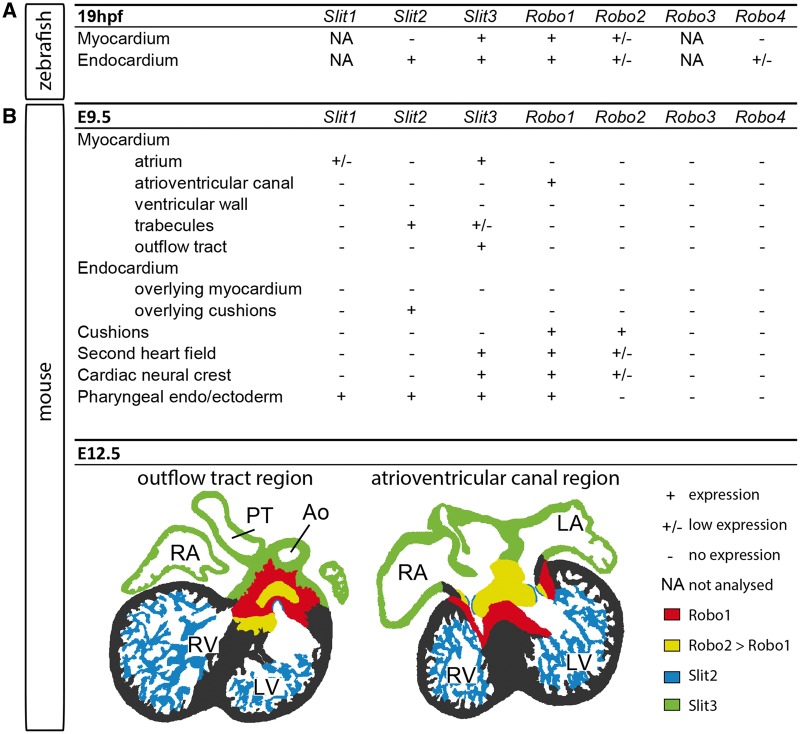
Expression patterns of the Slit and Robo genes during zebrafish and mouse heart development.^6–8^^,^^53^*A*, Expression of the *Slit* and *Robo* genes in the endocardium and myocardium at 19 h post-fertilization in zebrafish. *B*, Table of expression of the *Slit* and *Robo* genes in the different tissues at E9.5 in the mouse. *C*, Expression of the *Slit* and *Robo* genes at E12.5 in the mouse. The left section focuses on the outflow tract region, while the right section shows the atrioventricular region. Ao, aorta; PT, pulmonary trunk; RA, right atrium; RV, right ventricle; LA, left atrium; LV, left ventricle.

More evidence on the function of the Slit–Robo signalling pathway during heart development has come from zebrafish. In the zebrafish, after bilateral cardiac progenitor formation, endocardial cells start migrating towards the midline, slightly later followed by myocardial cells. When the bilateral endocardial cells and myocardial cells fuse at the midline, a cardiac disc is formed, which subsequently is transformed into a linear heart tube. The atrium and ventricle start to balloon out from the heart tube, to form the single atrium and ventricle of the adult fish heart.^69^^,^^70^ During cardiac progenitor cell migration (19 h post-fertilization), *slit2* is mainly expressed in endocardial cells, whereas *slit3* and *robo1* are observed more broadly in the myocardial and endocardial/endothelial cells. *Robo4* expression is sparsely detected in the endocardium, while *robo2* levels are very low in all these tissues (*Figure 3A*).^53^ Both *slit2* and *slit3* are still strongly expressed in the heart around the time of chamber formation.^71^

Using morpholinos, the different ligands and receptors were knocked down, revealing a similar requirement for the pathway in controlling cardiac cell migration and lumen formation as found in *Drosophila*. *Slit2* knock-down embryos show normal myocardial cell migration towards the midline. However, endocardial cell migration is disrupted; individual cells migrate faster with loss of directionality. Additionally, collective cell migration is disturbed, as individual migrating endocardial cells do not contact neighbouring cells and they extend numerous filopodia in multiple directions. *Slit2* morphant hearts develop multiple lumens,^53^ whereas in *slit2* gain-of-function embryos, endocardial cells do not form a disc but are located more diffusely, less densely packed at the midline. Endocardial cells also appear larger and less rounded than in control embryos. In contrast to *slit2* knock-down, *robo1* knock-down inhibits both endocardial and myocardial cell migration, resulting in unfused heart fields. Interestingly, also *robo1* gain-of-function results in incomplete heart field fusion. Endocardial cells in *robo1* morphants exhibit a rounded, non-migratory morphology. Migration of the heart fields to the midline is not delayed in *robo4* morphants, however, endocardial cell morphology and directionality are affected. At 48 hpf, fish with knock-down of *slit2*, *robo1*, and *robo4* show pericardial edema and circulation defects, without clear vascular patterning defects. The role of Slit3 seems more confined to the vasculature, as *slit3* morphants display highly penetrant vascular defects, including missing or detached intersomitic vessels and/or dorsal longitudinal anastomotic vessels. *Slit3* morphants do not show pericardial edema at 48 h post-fertilization but have not yet been analysed at earlier stages. In agreement with the very low levels of *robo2*, *robo2* homozygous knockout fish do not show defects in the cardiovascular system.^53^ These data suggest that Slit2, Robo1, and Robo4 are important for heart development and function in zebrafish, whereas Slit3 is mainly essential for vascular development. Intriguingly, both *slit2* and *slit3* have a miRNA encoded within an intron, mir218-1, and mir218-2, respectively. This miRNA is very well-conserved across species, from human and mouse to zebrafish and Xenopus,^72^ and has been shown to regulate Tbx5 expression in the heart.^71^ miR-218 is able to repress expression of both *robo1* and *robo2* mRNA.^53^^,^^71^^,^^72^ miR-218 knock-down in zebrafish results in a phenotype similar to *robo1* overexpression, suggesting functional regulation of Slit–Robo signalling by miR-218. Additionally, cross-talk was found between Robo1, Vegfa, and the Vegfr2 receptor to control heart field migration, indicating a Slit/miR-218/Robo/Vegf feedback regulatory loop regulating heart field migration.^53^

## 5. Disruption of Slit–Robo signalling in the mammalian heart affects multiple processes during heart development

Just as in *Drosophila* and zebrafish, formation of the mammalian heart starts by the migration of bilateral strips of cardiac progenitor cells to the midline, which happens around embryonic day (E) 7.5 in the mouse. The bilateral strips meet in the midline, where they fuse to form the heart tube. Cells are added to both poles of the heart to elongate the heart tube. Endocardial cells undergo endocardial-to-mesenchymal transformation to form the outflow tract and atrioventricular cushions. Subsequent expansion of the chambers, atrial, and ventricular septation and remodelling of the cardiac cushions into the valves and membranous septa will result in the septated four-chambered heart.^73^^,^^74^

Three *Slit* and the *Robo1*, *Robo2*, and *Robo4* genes display very distinct spatial and temporal expression patterns during mouse heart development. *Robo3* expression is mainly confined to the central nervous system and has not been detected in the heart at any stage during murine development,^6^^,^^75^ while *Robo4* is selectively expressed by coronary vessel, caval vein, aorta, and pulmonary trunk endothelial cells rather than endocardium.^7^*Slit1* expression is not observed at any stage in the developing heart after E9.5, although till E9.5 expression in the atria has been reported.^6–8^*Slit2* and *Slit3* on the other hand show distinct expression patterns in the heart all throughout development. From E8.5 to E9.5, *Slit2* is not observed in the myocardium but is strongly expressed in the pharyngeal region which is essential for the pharyngeal arch artery formation.^8^^,^^9^ Around E9.5–E10.5, strongest *Slit2* expression is detected in the ventricular trabecular myocardium but the ligand is also observed in parts of the second heart field, and the endocardium lining the outflow tract and atrioventricular cushions. Later, *Slit2* also becomes expressed in the epicardium, the aortic semilunar valves, and the mesenchyme surrounding the caval veins (*Figure 3B*).^6^^,^^7^ Of the *Slit* genes, *Slit3* is the earliest to be expressed in the developing heart, with detection at E7.5 in the cardiac crescent. At E8.5, *Slit3* is expressed in the ventral wall of the heart tube.^8^ By E9.5–E10.5, *Slit3* starts to show a similar expression pattern in the trabecular region of the ventricles to *Slit2*, albeit with much lower expression levels. In contrast to *Slit2*, *Slit3* is highly expressed in the myocardium, with presence in the outflow tract, atrial, and sinus horn myocardium including the sinus node. Furthermore, it is expressed in the cardiac neural crest, the second heart field, the tissues connecting the heart to the body, and later, the epicardium (*Figure 3B*).^6^^,^^7^*Slit3* has been reported to still be expressed in the adult ventricle.^72^

Of the two Robo receptors expressed in the developing mouse heart, Robo1 is more broadly distributed than Robo2. Both receptors are expressed in the venous pole of the linear heart tube at E8.5.^8^ At E9.5, *Robo1* expression is detected in the cardiac neural crest, second heart field, outflow tract and atrioventricular canal cushions, the myocardium of the atrioventricular canal and robustly in the mesenchyme surrounding the venous pole of the heart *Figure 3B*). When the cushions start maturing into the valves, *Robo1* expression is maintained in the atrioventricular and pulmonary semilunar valves, however, disappears from the aortic semilunar valves before birth. Furthermore, *Robo1* remains strongly expressed in the atrioventricular canal myocardium, which can now be recognized as the ventricular conduction system, including the atrioventricular node, the His bundle, and bundle branches.^6^^,^^7^ At E9.5–E10.5, *Robo2* expression is seen in a limited number of cardiac neural crest cells, and strongly in both the outflow tract and atrioventricular cushions (*Figure 3B*). *Robo2* expression has been reported to be expressed in the atria at E10.5^8^ but was later not detected in the myocardium at any stage during heart development (*Figure 3B*).^6^^,^^7^*Robo2* is still highly expressed in the pulmonary and aortic semilunar valves just before birth. These specific and partially overlapping expression patterns of the *Slit* and *Robo* genes in the developing heart indicate a large array of functions during heart development.

### 5.1 Slit–Robo signalling regulates cardiac neural crest and second heart field contribution to the heart

The initially formed primary heart tube is derived from progenitors called the ‘first heart field’, which will eventually form the left ventricle of the adult heart. After primary heart tube formation, cells are added to both the arterial and venous poles of the heart tube from a second heart field of progenitors. The second heart field, together with the neighbouring cardiac neural crest, are the main sources of cells contributing to the heart after primary heart tube formation. The second heart field contributes most of the cells to the right ventricle and atria, while contribution of the cardiac neural crest cells is important for septation of the aorta and pulmonary trunk as well as for proper development of the membranous ventricular septum.^76^ In the formation of the arterial pole of the heart, there is intricate signalling between the cardiac neural crest, the second heart field, and the neighbouring pharyngeal endoderm and ectoderm. Defects in this interplay often result in congenital heart disease. Intriguingly, both the cardiac neural crest and the second heart field cell populations express low levels of *Robo2* but high levels of *Slit3* and *Robo1*. *Slit2* is highly expressed in the surrounding pharyngeal endoderm and ectoderm, where it overlaps with *Slit3* expression.^7^^,^^9^ The components of the Slit–Robo pathway have been found to be regulated by a number of transcription factors in this region. Haploinsufficiency of *TBX1* causes DiGeorge Syndrome, which includes aortic arch patterning defects, conotruncal heart defects, and malformations of the thymus gland, parathyroid gland, and craniofacial structures.^77^ Homeobox-containing transcription factor Gbx2 acts downstream of T-box transcription factor Tbx1 to navigate cardiac neural crest cell migration. In both *Tbx1*^−^^/^^−^ mutants and *Gbx2*^−^^/^^−^ mutants, *Slit2* expression is diminished in the pharyngeal endoderm, whereas the number of *Robo1*-positive cardiac neural crest cells is reduced.^9^ These results suggest that the Robo1-expressing neural crest cells require Slit2 signalling from the surrounding tissues for normal development. Interestingly, both *Tbx1* and *Gbx2* mutants show abnormally organized endothelial cells,^78^ possibly linking back to the defects observed in *Drosophila* and zebrafish. As in *Drosophila* and zebrafish, in mouse, Robo1 and Robo4 have found to be involved in endothelial cell filopodia formation and cell motility.^79^ These data indicate a role for the Slit–Robo pathway in the developmental processes regulated by Tbx1 and Gbx2, suggesting its involvement downstream of TBX1 and GBX2 in DiGeorge syndrome.

### 5.2 Ventricular septal defects in absence of Slit–Robo signalling

**Figure 4:**
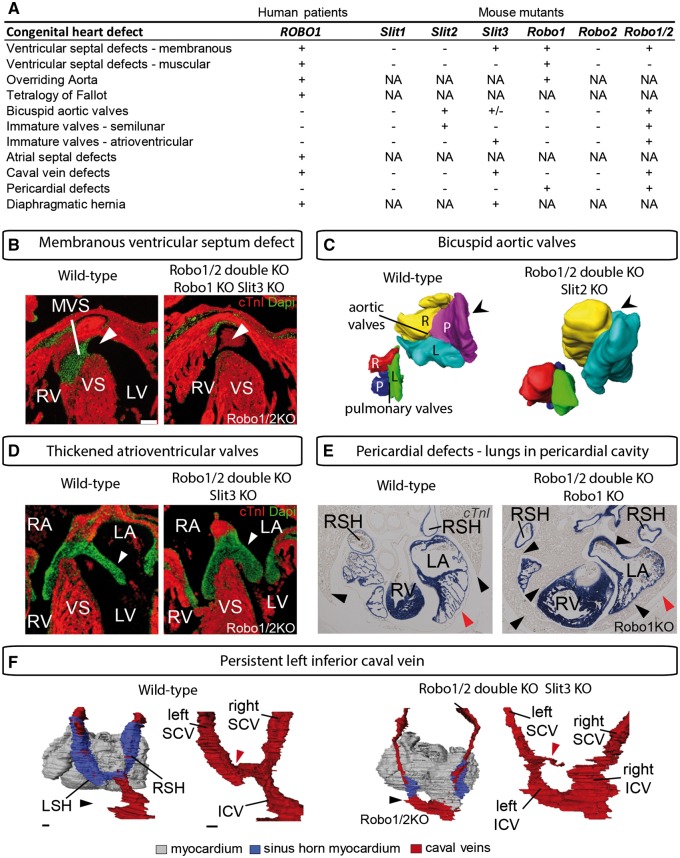
Heart defects found in patients and mouse mutants for the Slit and Robo genes, adapted from ^5–7^^,^^20^^,^^90^. *A*, Table showing the range of congenital heart defects identified in patients and mouse mutants. +, defect; −, no defect; ±, party affected; NA, not analysed. *B*, E14.5 wild-type and Robo1/Robo2 knockout heart showing the absence of the MVS in the knockout (arrow), which is also observed in the single Robo1 and Slit3 mutants. cTnI, cardiac troponin I, which labels the myocardium in red. Nuclear 4′,6-diamidino-2-phenylindole (DAPI) staining in green. MVS, membranous ventricular septum; VS, ventricular septum. *C*, 3D reconstruction of the semilunar valves of a wild-type and Robo1/Robo2 mutant at E15.5. R, right valve; L, left valve; P, posterior valve. The posterior aortic valve is missing in Robo1/Robo2 as well as Slit2 knockouts. *D*, Thickened immature atrioventricular valves (arrow) in the Robo1/2 double mutants at E15.5 and Slit3 mutants compared with wild-type littermates. *E*, Partial absence of the pericardium in Robo1 and Robo1/2 mutants. Red arrow indicates the location of the pericardium. In the mutant, the lungs completely envelop the heart (black arrows). LSH, left sinus horn; RSH, right sinus horn. F, dorsal view on the heart (grey) and caval veins (red) surrounded by sinus horn myocardium (blue) or only the caval veins of an E15.5 Robo1/2 double mutant and littermate control. Black arrow indicates the persistent left ICV in the mutant, which is also found in *Slit3* mutants. The mutant shows an abnormally small connection to the right atrium (red arrow). ICV, inferior caval vein; SCV, superior caval vein. Scale bars are 100 µm.

The membranous ventricular septum closes the communication between the right and left ventricles by fusion of the outflow tract cushions with the atrioventricular cushions and is normally completely closed in the mouse at E14.5. Loss of *Robo1* or both *Robo1* and *Robo2* results in membranous ventricular septum defects at birth, a defect also found in *Slit3*, but not in *Slit2* mutants (*Figure 4A* and *B*).^6^ Mice with an ENU-induced mutation in *Robo1* furthermore show double outlet right ventricle with membranous ventricular septal defects, muscular ventricular septal defects, and atrioventricular septal defects.^5^^,^^80^ Why the mice with ENU-induced *Robo1* mutation show a more severe phenotype than the full *Robo1* or *Robo1/2* mutants is not yet understood. The cause of the membranous septal defect in the *Slit* and *Robo* mutant mice is still not completely clear. Membranous ventricular septal defects can be caused by reduced contribution of cardiac neural crest or second heart field cells to the outflow tract or defects in endothelial-to-mesenchymal transformation and maturation of the cardiac cushions.^81^ All these processes are possibly affected in *Slit* and *Robo* mutant mice, with first, reduced outflow tract cushion closure in the part of the cushions that is neural crest derived. Second, there is delayed maturation of the cardiac cushions. Third, the strong expression of *Robo1* as well as *Slit2* and *3* in the second heart field also suggests a role in the second heart field, although this will still need to be further examined.^6^ The aorta and pulmonary trunk are normally separated in all mutants. However, in both the *Robo1* and double *Robo* mutant, the outflow tract vessels are slightly less rotated than normal, with the aorta slightly more to the right of the pulmonary trunk.

### 5.3 Early cardiac chamber formation requires repression of Slit3 by Nkx2-5 and Tbx2

The cardiac chambers locally balloon out from the primitive heart tube.^82^ Transcription factors Gata4, Nkx2-5, Tbx20, and transcriptional activator Tbx5 are expressed throughout most of the heart tube, and interact to activate the chamber formation program. However, in the atrioventricular canal, where transcriptional repressors Tbx2 and Tbx3 are present and can bind instead of Tbx5, chamber formation is repressed. This area will largely form the cardiac conduction system.^82^ These transcription factors are some of the genes most frequently screened for mutations in congenital heart disease, causing ventricular, atrial, and atrioventricular septal defects, as well as a range of outflow tract defects.^2^^,^^82^^,^^83^ Although data on Slit2 is largely lacking, Slit3 expression seems tightly interlinked with these genes. In absence of *Nkx2-5*, *Slit3* expression expands throughout the entire heart tube at E8.5, whereas *Robo2* expression is absent. In contrast, when *Tbx20* is absent, *Slit3* expression completely disappears from the heart.^8^ The same phenotype is observed in hearts overexpressing *Tbx2*, whereas when *Tbx2* is knocked out, *Slit3* expression is activated in the atrioventricular canal. In vitro assays indicate that Tbx2 can directly bind to *Slit3*,^8^ together suggesting that *Slit3* expression is restricted to the ventral wall of the E8.5 heart tube by Nkx2-5 and excluded from the atrioventricular canal by Tbx2. These data indicate a role for the Slit–Robo pathway during early heart patterning.

*CDH7* mutations are linked to CHARGE syndrome, which is characterized by a specific pattern of defects, including ocular coloboma, heart malformations, atresia of the choanae, growth retardation, genital hypoplasia, and ear abnormalities.^10^ Both *Slit2* and *Robo2* expression is reduced in heart-specific mutants for *Cdh7*, suggesting possible involvement of the pathway in the development of CHARGE sysndrome.^10^

### 5.4 Slit and Robo mutants display a spectrum of valve malformations

The mesenchymal cushions lining the early heart tube will remodel to form the semilunar aortic and pulmonary outflow tract valves as well as the mitral and tricuspid atrioventricular valves.^84^*Robo1/Robo2* double mutants have thickened immature semilunar and atrioventricular valves as well as highly penetrant bicuspid aortic valves (*Figure 4A, C*, and *D*).^6^ Bicuspid aortic valves only have two complete leaflets, while the third leaflet is either absent or incomplete. Bicuspid aortic valves are among the most common of congenital defects, affecting around 1–2% of the population.^1^^,^^85^ Although *Slit2* mutants have normal atrioventricular valves, these mutants do display bicuspid aortic valves, albeit with lower penetrance than in the *Robo1/Robo2* double mutants. In contrast, *Slit3* mutants have clearly thickened atrioventricular valves. The posterior non-coronary aortic valve is hypoplastic in *Slit3* mutants but never absent (*Figure 4A, C*, and *D*).^6^ NOTCH1 is one of the few transcriptional regulators linked to bicuspid aortic valve disease in humans to date.^86^ The different genes of the Slit–Robo and Notch-Hey/Hes pathways have very similar overlapping expression patterns during heart development.^6^^,^^87^ As the expression of *Notch*- and downstream *Hey* and *Hes* genes is down-regulated in *Robo1* mutants, reduced Notch signalling might underlie the valve defects in these mice.^6^^,^^88^ During cortical development, it has been found that the intracellular domain of Robo2 is able to bind directly to Notch1 target *Hes1*,^89^ however, *Notch* expression is down-regulated in the absence of *Robo* in the heart, suggesting that Robo might activate *Notch* expression instead of directly regulating Notch-responsive genes.^6^ Its role during valve formation is another important role for the Slit–Robo signalling pathway during heart development.

### 5.5 Loss of Robo1 results in partial absence of the pericardium

Pericardial (pleuropericardial membrane) defects can involve the entire pericardium, or be partial, with the majority affecting the left side of the pericardium rather than the right. This congenital defect is mostly asymptomatic and is often discovered incidental. In case of partial absence, herniation of the heart through the defect can lead to obstruction of blood flow through the heart.^11^ Fifty percentage of mice lacking the Robo1 receptor and 70% of mice lacking both the Robo1 and Robo2 receptor show partial absence of the pericardial, in particular the part of the membrane between the superior caval veins is consistently missing (*Figure 4E*).^7^ As a result, the lungs penetrate through the hole into the pericardial cavity and completely envelop the heart. This defect is not recapitulated in *Slit2* and *Slit3* mutants, suggesting functional redundancy of the ligands in this process.^7^ However, congenital diaphragmatic hernias have been identified in absence of *Slit3*,^20^^,^^90^ indicating that Slit3 is important for the division of the coelomic cavities. One of the patients with a loss of function variant in *ROBO1* was diagnosed with congenital diphragmatic hernia.^5^ In human, diaphragmatic hernias coexist with pericardium defects, suggesting a related developmental mechanism or involvement of the same gene pathways.^11^ Associated cardiac anomalies are seen in 30% of all pericardial defect cases, including septal defects, patent ductus arteriosus, bicuspid aortic valves, and anomalous inferior caval veins.^11–13^ Although no causative genes have been identified yet in patients, the combination of defects points to the *SLIT3*, *ROBO1*, and *ROBO2* genes as likely candidates. Very little is known on how the pericardial defects develop and which molecular pathways are involved. In *Robo1* mutants, this seems to be caused by the fact that the cranial pericardial cavity expands too far dorsally and the caval veins do not become localized normally within the cavity. The neural crest cells present dorsally of the cranial pericardial cavity show reduced adhesion and increased cell death, likely allowing the pericardial cavity to extend into the region normally occupied by neural crest cells. As a result, the lungs are forced to develop ventral to the caval veins, indirectly blocking the closure of the pericardial membranes.^7^

### 5.6 Persistent left inferior caval veins in Slit3 and Robo1 Robo2 double mutants

One of the main roles of the Slit–Robo pathway during *Drosophila* and zebrafish heart development is its function during cardiac lumen formation.^53^^,^^65^ Although this has not been investigated sufficiently, so far, similar defects in cardiac lumen formation have not yet been identified in mouse mutants for the *Slit* and *Robo* genes. However, lumen formation of the caval veins is affected to varying degrees in these mutants. Although the caval veins develop too far dorsally in *Robo1* mutants, and enter the pericardial cavity more caudally, they are relatively normal and surrounded by sinus horn myocardium near the entrance to the atrium. However, additional absence of *Robo2* or absence of *Slit3* results in severely malformed and thin caval veins (*Figure 4F*).^7^ If this strongly reduced vein lumen develops through a similar mechanism as during *Drosophila* and zebrafish heart development is yet to be determined. The connection of the left caval vein to the right atrium is abnormally small or only connecting to the coronary circulation, with minimal sinus horn myocardium development. The sinus node, which largely forms within the sinus horn myocardium, has a slightly different morphology but has a normal molecular signature. Additionally, the double *Robo* and *Slit3* mutants display persistent left inferior caval veins, which join the right inferior caval vein at liver level. These defects are not present in *Slit2* mutants.^7^ Although the left superior caval veins always persists in mouse, it normally regresses in human to become the coronary sinus, however, a left superior caval vein was identified in a patient with a loss of function *ROBO* variant.^5^ Based on our knowledge from *Drosophila* and zebrafish, a role for the pathway in the endothelium is expected during early caval vein lumen formation. However, this has not been studied yet in detail and our knowledge on how these caval vein defects develop is limited. Endothelial cells require Slit2–Robo4 interaction for stabilizing the vasculature during angiogenesis,^34^ indicating that further research is necessary at much earlier stages of caval vein development.

## 6. Conclusions and future directions

The range of different defects observed between the various *Slit* and *Robo* mutants indicates the requirement of several specific combinations of ligand–receptor interactions during different stages of development and in different parts of the mammalian heart. For example, Slit3–Robo1 interaction seems most important during development of the membranous ventricular septum, while Slit2 binding to both Robo1 and Robo2 is most important for the presence of all three aortic semilunar valve leaflets. The fact that, for example, pericardial defects are not observed in any of the *Slit* mutants, suggests functional redundancy of these ligands. The presence of three Slit ligands and four Robo receptors in mammals might also explain the less severe phenotype observed in single *Slit* mouse mutants, compared with the severe early hearts defects observed in *Drosophila* that only has one Slit ligand, eventhough triple *Slit* mutants survive till at least E12.5.^91^ Although we now have an extensive overview of the role of Slit–Robo signalling during the many different aspects of heart development, our knowledge on how the different cell types interact during this process is very limited. Especially in mouse, all research so far has been performed using full constitutive knock outs, and further research using tissue-specific mutants will allow to dissect out the different source and responsive tissues in the different parts of the heart. The role of the pathway during *Drosophila* heart development has been studied on a more detailed cellular level and it will be important to extrapolate these findings to mouse and human. In particular, the aetiology of the caval vein defects requires studying earlier stages of vessel development. Further study of the identified range of valve defects, and in particular the already early in development recognisable bicuspid aortic valves, might help understand the aetiology of common congenital valve defects found in patients. The pathway has been studied most extensively during axon guidance and it will be interesting to study a role of the pathway during the development of the innervation of the heart.

## References

[1] Hoffman JI, Kaplan S. The incidence of congenital heart disease. J Am Coll Cardiol 2002; 39:1890–1900. 12084585 10.1016/s0735-1097(02)01886-7

[2] Bruneau BG. The developmental genetics of congenital heart disease. Nature 2008; 451:943–948. 18288184 10.1038/nature06801

[3] Gelb B, Brueckner M, Chung W, Goldmuntz E, Kaltman J, Kaski JP, Kim R, Kline J, Mercer-Rosa L, Porter G, Roberts A, Rosenberg E, Seiden H, Seidman C, Sleeper L, Tennstedt S, Schramm C, Burns K, Pearson G, Breitbart R, Colan S, Geva J, Monafo A, Stryker J, McDonough B, Seidman J, Edman S, Garbarini J, Hakonarson H, Mitchell L. The congenital heart disease genetic network study: rationale, design, and early results. Circ Res 2013; 112:698–706. 23410879 10.1161/CIRCRESAHA.111.300297 PMC3679175

[4] Zaidi S, Brueckner M. Genetics and genomics of congenital heart disease. Circ Res 2017; 120:923–940. 28302740 10.1161/CIRCRESAHA.116.309140 PMC5557504

[5] Kruszka P, Tanpaiboon P, Neas K, Crosby K, Berger SI, Martinez AF, Addissie YA, Pongprot Y, Sittiwangkul R, Silvilairat S, Makonkawkeyoon K, Yu L, Wynn J, Bennett JT, Mefford HC, Reynolds WT, Liu X, Mommersteeg MTM, Chung WK, Lo C, Muenke M. Loss of function in ROBO1 is associated with tetralogy of Fallot and septal defects. J Med Genet 2017; 54:825–829. 28592524 10.1136/jmedgenet-2017-104611

[6] Mommersteeg MTM, Yeh ML, Parnavelas JG, Andrews WD. Disrupted Slit-Robo signalling results in membranous ventricular septum defects and bicuspid aortic valves. Cardiovasc Res 2015; 106:55–66. 25691540 10.1093/cvr/cvv040 PMC4362403

[7] Mommersteeg MTM, Andrews WD, Ypsilanti AR, Zelina P, Yeh ML, Norden J, Kispert A, Chédotal A, Christoffels VM, Parnavelas JG, Chedotal A. Slit-Roundabout signaling regulates the development of the cardiac systemic venous return and pericardium. Circ Res 2013; 112:465–475. 23255421 10.1161/CIRCRESAHA.112.277426

[8] Medioni C, Bertrand N, Mesbah K, Hudry B, Dupays L, Wolstein O, Washkowitz AJ, Papaioannou VE, Mohun TJ, Harvey RP, Zaffran S. Expression of Slit and Robo genes in the developing mouse heart. Dev Dyn 2010; 239:3303–3311. 20941780 10.1002/dvdy.22449 PMC2996720

[9] Calmont A, Ivins S, Bueren KLV, Papangeli I, Kyriakopoulou V, Andrews WD, Martin JF, Moon AM, Illingworth EA, Basson MA, Scambler PJ. Tbx1 controls cardiac neural crest cell migration during arch artery development by regulating Gbx2 expression in the pharyngeal ectoderm. Development 2009; 136:3173–3183. 19700621 10.1242/dev.028902 PMC2730371

[10] Payne S, Burney MJ, McCue K, Popal N, Davidson SM, Anderson RH, Scambler PJ. A critical role for the chromatin remodeller CHD7 in anterior mesoderm during cardiovascular development. Dev Biol 2015; 405:82–95. 26102480 10.1016/j.ydbio.2015.06.017 PMC4534312

[11] Faridah Y, Julsrud PR. Congenital absence of pericardium revisited. Int J Cardiovasc Imaging 2002; 18:67–73. 12135126 10.1023/a:1014350814067

[12] Castro EC, Devine W, Galambos C. The anatomy of a novel malformation of the cardinal vein system. Pediatr Dev Pathol 2010; 13:318–321. 19863444 10.2350/09-07-0671-CR.1

[13] Matsuoka T, Kimura F, Sugiyama K, Nagata N, Takatani O. Anomalous inferior vena cava with azygos continuation, dysgenesis of lung, and clinically suspected absence of left pericardium. Chest 1990; 97:747–749. 2306978 10.1378/chest.97.3.747

[14] Jürgens G, Wieschaus E, Nüsslein-Volhard C, Kluding H. Mutations affecting the pattern of the larval cuticle in *Drosophila melanogaster*. Wilehm Roux Arch Dev Biol 1984;193:283–295. 28305338 10.1007/BF00848157

[15] Rothberg JM, Hartley DA, Walther Z, Artavanis-Tsakonas S. Slit: an EGF-homologous locus of *D. melanogaster* involved in the development of the embryonic central nervous system. Cell 1988;55:1047–1059. 3144436 10.1016/0092-8674(88)90249-8

[16] Kidd T, Bland KS, Goodman CS. Slit is the midline repellent for the Robo receptor in Drosophila. Cell 1999;96:785–794. 10102267 10.1016/s0092-8674(00)80589-9

[17] Kidd T, Brose K, Mitchell KJ, Fetter RD, Tessier-Lavigne M, Goodman CS, Tear G. Roundabout controls axon crossing of the CNS midline and defines a novel subfamily of evolutionarily conserved guidance receptors. Cell 1998; 92:205–215. 9458045 10.1016/s0092-8674(00)80915-0

[18] Seeger M, Tear G, Ferres-Marco D, Goodman CS. Mutations affecting growth cone guidance in Drosophila: genes necessary for guidance toward or away from the midline. Neuron 1993;10:409–426. 8461134 10.1016/0896-6273(93)90330-t

[19] Piper M, Georgas K, Yamada T, Little M. Expression of the vertebrate Slit Gene family and their putative receptors, the Robo genes, in the developing murine kidney. Mech Dev 2000; 94:213–217. 10842075 10.1016/s0925-4773(00)00313-0

[20] Yuan W, Rao Y, Babiuk RP, Greer JJ, Wu JY, Ornitz DM. A genetic model for a central (septum transversum) congenital diaphragmatic hernia in mice lacking Slit3. Proc Natl Acad Sci U S A 2003; 100:5217–5222. 12702769 10.1073/pnas.0730709100 PMC154325

[21] Ypsilanti AR, Zagar Y, Chedotal A. Moving away from the midline: new developments for Slit and Robo. Development 2010; 137:1939–1952. 20501589 10.1242/dev.044511

[22] Seiradake E, Philipsborn ACV, Henry M, Fritz M, Lortat-Jacob H, Jamin M, Hemrika W, Bastmeyer M, Cusack S, McCarthy AA. Structure and functional relevance of the Slit2 homodimerization domain. EMBO Rep 2009; 10:736–741. 19498462 10.1038/embor.2009.95 PMC2693880

[23] Brose K, Bland KS, Wang KH, Arnott D, Henzel W, Goodman CS, Tessier-Lavigne M, Kidd T. Slit proteins bind Robo receptors and have an evolutionarily conserved role in repulsive axon guidance. Cell 1999; 96:795–806. 10102268 10.1016/s0092-8674(00)80590-5

[24] Liang Y, Annan RS, Carr SA, Popp S, Mevissen M, Margolis RK, Margolis RU. Mammalian homologues of the Drosophila slit protein are ligands of the heparan sulfate proteoglycan glypican-1 in brain. J Biol Chem 1999;274:17885–17892. 10364234 10.1074/jbc.274.25.17885

[25] Johnson KG, Ghose A, Epstein E, Lincecum J, O’Connor MB, Van Vactor D. Axonal heparan sulfate proteoglycans regulate the distribution and efficiency of the repellent Slit during midline axon guidance. Curr Biol 2004; 14:499–504. 15043815 10.1016/j.cub.2004.02.005

[26] Steigemann P, Molitor A, Fellert S, Jäckle H, Vorbrüggen G. Heparan sulfate proteoglycan syndecan promotes axonal and myotube guidance by Slit/Robo signaling. Curr Biol 2004; 14:225–230. 14761655 10.1016/j.cub.2004.01.006

[27] Xiao T, Staub W, Robles E, Gosse NJ, Cole GJ, Baier H. Assembly of lamina-specific neuronal connections by Slit bound to type IV collagen. Cell Elsevier Inc 2011; 146:164–176. 10.1016/j.cell.2011.06.016 PMC3136219 21729787

[28] Wright KM, Lyon KA, Leung H, Leahy DJ, Ma L, Ginty DD. Dystroglycan organizes axon guidance cue localization and axonal pathfinding. Neuron 2012; 76:931–944. 23217742 10.1016/j.neuron.2012.10.009 PMC3526105

[29] Dascenco D, Erfurth ML, Izadifar A, Song M, Sachse S, Bortnick R, Urwyler O, Petrovic M, Ayaz D, He H, Kise Y, Thomas F, Kidd T, Schmucker D. Slit and receptor tyrosine phosphatase 69D confer spatial specificity to axon branching via Dscam1. Cell Elsevier Inc 2015; 162:1140–1154. 10.1016/j.cell.2015.08.003 PMC4699798 26317474

[30] Fujisawa K, Wrana JL, Culotti JG. The Slit receptor EVA-1 coactivates a SAX-3/Robo–mediated guidance signal in *C. elegans*. Science 2007;317:1934. 17901337 10.1126/science.1144874

[31] James G, Foster SR, Key B, Beverdam A. The expression pattern of EVA1C, a novel Slit receptor, is consistent with an axon guidance role in the mouse nervous system. PLoS One 2013;8:e74115. 24040182 10.1371/journal.pone.0074115 PMC3767613

[32] Nguyen Ba-Charvet KT, Brose K, Ma L, Wang KH, Marillat V, Sotelo C, Tessier-Lavigne M, Chédotal A. Diversity and specificity of actions of Slit2 proteolytic fragments in axon guidance. J Neurosci 2001; 21:4281–4289. 11404413 10.1523/JNEUROSCI.21-12-04281.2001 PMC6762758

[33] Delloye-Bourgeois C, Jacquier A, Charoy C, Reynaud F, Nawabi H, Thoinet K, Kindbeiter K, Yoshida Y, Zagar Y, Kong Y, Jones YE, Falk J, Chédotal A, Castellani V. PlexinA1 is a new Slit receptor and mediates axon guidance function of Slit C-terminal fragments. Nat Neurosci 2015; 18:36–45. 25485759 10.1038/nn.3893

[34] Jones CA, London NR, Chen H, Park KW, Sauvaget D, Stockton RA, Wythe JD, Suh W, Larrieu-Lahargue F, Mukouyama Y-S, Lindblom P, Seth P, Frias A, Nishiya N, Ginsberg MH, Gerhardt H, Zhang K, Li DY. Robo4 stabilizes the vascular network by inhibiting pathologic angiogenesis and endothelial hyperpermeability. Nat Med 2008; 14:448–453. 18345009 10.1038/nm1742 PMC2875252

[35] Dickson BJ, Gilestro GF. Regulation of commissural axon pathfinding by Slit and its Robo receptors. Annu Rev Cell Dev Biol 2006; 22:651–675. 17029581 10.1146/annurev.cellbio.21.090704.151234

[36] Clark K, Hammond E, Rabbitts P. Temporal and spatial expression of two isoforms of the Dutt1/Robo1 gene in mouse development. FEBS Lett 2002; 523:12–16. 12123796 10.1016/s0014-5793(02)02904-6

[37] Chen Z, Gore BB, Long H, Ma L, Tessier-Lavigne M. Alternative splicing of the Robo3 axon guidance receptor governs the midline switch from attraction to repulsion. Neuron 2008; 58:325–332. 18466743 10.1016/j.neuron.2008.02.016

[38] Dalkic E, Kuscu C, Sucularli C, Aydin IT, Akcali KC, Konu O. Alternatively spliced Robo2 isoforms in zebrafish and rat. Dev Genes Evol 2006; 216:555–563. 16625395 10.1007/s00427-006-0070-y

[39] Barak R, Lahmi R, Gevorkyan-Airapetov L, Levy E, Tzur A, Opatowsky Y. Crystal structure of the extracellular juxtamembrane region of Robo1. J Struct Biol 2014; 186:283–291. 24607414 10.1016/j.jsb.2014.02.019

[40] Coleman HA, Labrador J-P, Chance RK, Bashaw GJ. The Adam family metalloprotease Kuzbanian regulates the cleavage of the Roundabout receptor to control axon repulsion at the midline. Development 2010; 137:2417–2426. 20570941 10.1242/dev.047993 PMC2889607

[41] Seki M, Watanabe A, Enomoto S, Kawamura T, Ito H, Kodama T, Hamakubo T, Aburatani H. Human ROBO1 is cleaved by metalloproteinases and γ-secretase and migrates to the nucleus in cancer cells. FEBS Lett 2010; 584:2909–2915. 20471383 10.1016/j.febslet.2010.05.009

[42] Morlot C, Thielens NM, Ravelli RBG, Hemrika W, Romijn RA, Gros P, Cusack S, McCarthy AA. Structural insights into the Slit-Robo complex. Proc Natl Acad Sci U S A 2007; 104:14923–14928. 17848514 10.1073/pnas.0705310104 PMC1975871

[43] Liu Z, Patel K, Schmidt H, Andrews W, Pini A, Sundaresan V. Extracellular Ig domains 1 and 2 of Robo are important for ligand (Slit) binding. Mol Cell Neurosci 2004; 26:232–240. 15207848 10.1016/j.mcn.2004.01.002

[44] Zhang F, Moniz HA, Walcott B, Moremen KW, Linhardt RJ, Wang L. Characterization of the interaction between Robo1 and heparin and other glycosaminoglycans. Biochimie 2013; 95:2345–2353. 23994753 10.1016/j.biochi.2013.08.018 PMC3871176

[45] Evans TA, Santiago C, Arbeille E, Bashaw GJ. Robo2 acts in trans to inhibit Slit-Robo1 repulsion in pre-crossing commissural axons. Elife 2015; 4:1–26. 10.7554/eLife.08407 PMC4505356 26186094

[46] Hivert B, Liu Z, Chuang C-Y, Doherty P, Sundaresan V. Robo1 and Robo2 are homophilic binding molecules that promote axonal growth. Mol Cell Neurosci 2002; 21:534–545. 12504588 10.1006/mcne.2002.1193

[47] Alavi M, Song M, King GLA, Gillis T, Propst R, Lamanuzzi M, Bousum A, Miller A, Allen R, Kidd T. Dscam1 forms a complex with Robo1 and the N-terminal fragment of Slit to promote the growth of longitudinal axons. PLoS Biol 2016; 14:e1002560–e1002531. 27654876 10.1371/journal.pbio.1002560 PMC5031454

[48] Stein E, Tessier-Lavigne M. Hierarchical organization of guidance receptors: silencing of netrin attraction by Slit through a Robo/DCC receptor complex. Science 2001;291:1928. 11239147 10.1126/science.1058445

[49] Prasad A, Qamri Z, Wu J, Ganju RK. Slit-2/Robo-1 modulates the CXCL12/CXCR4-induced chemotaxis of T cells. J Leukoc Biol 2007; 82:465–476. 17565045 10.1189/jlb.1106678 PMC2286829

[50] Banerjee S, Blauth K, Peters K, Rogers SL, Fanning AS, Bhat MA. Drosophila neurexin IV interacts with Roundabout and is required for repulsive midline axon guidance. J Neurosci 2010;30:5653–5667. 20410118 10.1523/JNEUROSCI.6187-09.2010 PMC2869042

[51] Hernández-Miranda LR, Cariboni A, Faux C, Ruhrberg C, Cho JH, Cloutier J-F, Eickholt BJ, Parnavelas JG, Andrews WD. Robo1 regulates semaphorin signaling to guide the migration of cortical interneurons through the ventral forebrain. J Neurosci 2011; 31:6174–6187. 21508241 10.1523/JNEUROSCI.5464-10.2011 PMC3088089

[52] Macias H, Moran A, Samara Y, Moreno M, Compton JE, Harburg G, Strickland P, Hinck L. SLIT/ROBO1 signaling suppresses mammary branching morphogenesis by limiting basal cell number. Dev Cell 2011; 20:827–840. 21664580 10.1016/j.devcel.2011.05.012 PMC3129866

[53] Fish JE, Wythe JD, Xiao T, Bruneau BG, Stainier DYR, Srivastava D, Woo S. A Slit/miR-218/Robo regulatory loop is required during heart tube formation in zebrafish. Development 2011; 138:1409–1419. 21385766 10.1242/dev.060046 PMC3050667

[54] Dubrac A, Genet G, Ola R, Zhang F, Pibouin-Fragner L, Han J, Zhang J, Thomas JL, Chedotal A, Schwartz MA, Eichmann A. Targeting NCK-mediated endothelial cell front-rear polarity inhibits neovascularization. Circulation 2016; 133:409–421. 26659946 10.1161/CIRCULATIONAHA.115.017537 PMC4729599

[55] Wong K, Ren XR, Huang YZ, Xie Y, Liu G, Saito H, Tang H, Wen L, Brady-Kalnay SM, Mei L, Wu JY, Xiong WC, Rao Y. Signal transduction in neuronal migration: roles of GTPase activating proteins and the small GTPase Cdc42 in the Slit-Robo pathway. Cell 2001; 107:209–221. 11672528 10.1016/s0092-8674(01)00530-x

[56] Round JE, Sun H. The adaptor protein Nck2 mediates Slit1-induced changes in cortical neuron morphology. Mol Cell Neurosci 2011; 47:265–273. 21600986 10.1016/j.mcn.2011.04.009

[57] Yang L, Bashaw GJ. Son of sevenless directly links the Robo receptor to rac activation to control axon repulsion at the midline. Neuron 2006; 52:595–607. 17114045 10.1016/j.neuron.2006.09.039

[58] Battye R, Stevens A, Jacobs JR. Axon repulsion from the midline of the Drosophila CNS requires Slit function. Development 1999;126:2475–2481. 10226006 10.1242/dev.126.11.2475

[59] Rotstein B, Paululat A. On the morphology of the Drosophila heart. J Cardiovasc Dev Dis 2016;3:15. 10.3390/jcdd3020015 PMC5715677 29367564

[60] Bier E. Drosophila, the golden bug, emerges as a tool for human genetics. Nat Rev Genet 2005;6:9–23. 15630418 10.1038/nrg1503

[61] Tao Y, Schulz R A. Heart development in Drosophila. Semin Cell Dev Biol 2007;18:3–15. 17208472 10.1016/j.semcdb.2006.12.001

[62] MacMullin A, Jacobs JR. Slit coordinates cardiac morphogenesis in Drosophila. Dev Biol 2006;293:154–164. 16516189 10.1016/j.ydbio.2006.01.027

[63] Qian L, Liu J, Bodmer R. Slit and Robo control cardiac cell polarity and morphogenesis. Curr Biol 2005; 15:2271–2278. 16360689 10.1016/j.cub.2005.10.037

[64] Santiago-Martinez E, Soplop NH, Kramer SG, Santiago-Martínez E, Soplop NH, Kramer SG, Santiago-Martinez E, Soplop NH, Kramer SG. Lateral positioning at the dorsal midline: Slit and Roundabout receptors guide Drosophila heart cell migration. Proc Natl Acad Sci U S A 2006;103:12441–12446. 16888037 10.1073/pnas.0605284103 PMC1567898

[65] Santiago-Martínez E, Soplop NH, Patel R, Kramer SG, Santiago-Martinez E, Soplop NH, Patel R, Kramer SG, Santiago-Martínez E, Soplop NH, Patel R, Kramer SG. Repulsion by Slit and Roundabout prevents Shotgun/E-cadherin-mediated cell adhesion during Drosophila heart tube lumen formation. J Cell Biol 2008;182:241–248. 18663139 10.1083/jcb.200804120 PMC2483515

[66] Harpaz N, Ordan E, Ocorr K, Bodmer R, Volk T. Multiplexin promotes heart but not aorta morphogenesis by polarized enhancement of Slit/Robo activity at the heart lumen. PLoS Genet 2013; 9:e1003597. 23825967 10.1371/journal.pgen.1003597 PMC3694841

[67] Medioni C, Astier M, Zmojdzian M, Jagla K, Sémériva M, Semeriva M. Genetic control of cell morphogenesis during *Drosophila melanogaster* cardiac tube formation. J Cell Biol 2008;182:249–261. 18663140 10.1083/jcb.200801100 PMC2483531

[68] Zmojdzian M, Ponte JPD, Jagla K. Cellular components and signals required for the cardiac outflow tract assembly in Drosophila. Proc Natl Acad Sci U S A 2008;105:2475–2480. 18250318 10.1073/pnas.0706402105 PMC2268161

[69] Bakkers J. Zebrafish as a model to study cardiac development and human cardiac disease. Cardiovasc Res 2011; 91:279–288. 21602174 10.1093/cvr/cvr098 PMC3125074

[70] Staudt D, Stainier D. Uncovering the molecular and cellular mechanisms of heart development using the zebrafish. Annu Rev Genet 2012; 46:397–418. 22974299 10.1146/annurev-genet-110711-155646 PMC6982417

[71] Chiavacci E, Dolfi L, Verduci L, Meghini F, Gestri G, Evangelista AMM, Wilson SW, Cremisi F, Pitto L. MicroRNA 218 mediates the effects of Tbx5a over-expression on zebrafish heart development. PLoS One 2012;7:e50536. 23226307 10.1371/journal.pone.0050536 PMC3511548

[72] Small EM, Sutherland LB, Rajagopalan KN, Wang S, Olson EN. Microrna-218 regulates vascular patterning by modulation of Slit-Robo signaling. Circ Res 2010; 107:1336–1344. 20947829 10.1161/CIRCRESAHA.110.227926 PMC2997642

[73] Moorman AF, Christoffels VM. Cardiac chamber formation: development, genes and evolution. Physiol Rev 2003; 83:1223–1267. 14506305 10.1152/physrev.00006.2003

[74] Buckingham M, Meilhac S, Zaffran S. Building the mammalian heart from two sources of myocardial cells. Nat Rev Genet 2005; 6:826–837. 16304598 10.1038/nrg1710

[75] Camurri L, Mambetisaeva E, Sundaresan V. Rig-1 a new member of Robo family genes exhibits distinct pattern of expression during mouse development. Gene Expr Patterns 2004; 4:99–103. 14678835 10.1016/s1567-133x(03)00142-x

[76] Scholl AMM, Kirby MLL. Signals controlling neural crest contributions to the heart. Wiley Interdiscip Rev Syst Biol Med 2009; 1:220–227. 20490374 10.1002/wsbm.8 PMC2873602

[77] Papangeli I, Scambler P. The 22q11 deletion: DiGeorge and velocardiofacial syndromes and the role of TBX1. Wiley Interdiscip Rev Dev Biol 2013; 2:393–403. 23799583 10.1002/wdev.75

[78] Byrd NA, Meyers EN. Loss of Gbx2 results in neural crest cell patterning and pharyngeal arch artery defects in the mouse embryo. Dev Biol 2005; 284:233–245. 15996652 10.1016/j.ydbio.2005.05.023

[79] Sheldon H, Andre M, Legg JA, Heal P, Herbert JM, Sainson R, Sharma AS, Kitajewski JK, Heath VL, Bicknell R. Active involvement of Robo1 and Robo4 in filopodia formation and endothelial cell motility mediated via WASP and other actin nucleation-promoting factors. FASEB J 2009; 23:513–522. 18948384 10.1096/fj.07-098269 PMC4048916

[80] Li Y, Klena NT, Gabriel GC, Liu X, Kim AJ, Lemke K, Chen Y, Chatterjee B, Devine W, Damerla RR, Chang C, Yagi H, San Agustin JT, Thahir M, Anderton S, Lawhead C, Vescovi A, Pratt H, Morgan J, Haynes L, Smith CL, Eppig JT, Reinholdt L, Francis R, Leatherbury L, Ganapathiraju MK, Tobita K, Pazour GJ, Lo CW. Global genetic analysis in mice unveils central role for cilia in congenital heart disease. Nature 2015; 521:520–524. 25807483 10.1038/nature14269 PMC4617540

[81] Neeb Z, Lajiness JD, Bolanis E, Conway SJ. Cardiac outflow tract anomalies. Wiley Interdiscip Rev Dev Biol 2013; 2:499–530. 24014420 10.1002/wdev.98 PMC4021394

[82] Rana MS, Christoffels VM, Moorman AFM. A molecular and genetic outline of cardiac morphogenesis. Acta Physiol 2013; 207:588–615. 10.1111/apha.12061 23297764

[83] Fahed AC, Gelb BD, Seidman JG, Seidman CE. Genetics of congenital heart disease: the glass half empty. Circ Res 2013; 112:707–720. 23410880 10.1161/CIRCRESAHA.112.300853 PMC3827691

[84] Combs MD, Yutzey KE. Heart valve development: regulatory networks in development and disease. Circ Res 2009; 105:408–421. 19713546 10.1161/CIRCRESAHA.109.201566 PMC2777683

[85] Siu SC, Silversides CK. Bicuspid aortic valve disease. J Am Coll Cardiol 2010; 55:2789–2800. 20579534 10.1016/j.jacc.2009.12.068

[86] McKellar SH, Tester DJ, Yagubyan M, Majumdar R, Ackerman MJ, Sundt TM III. Novel NOTCH1 mutations in patients with bicuspid aortic valve disease and thoracic aortic aneurysms. J Thorac Cardiovasc Surg 2007;134:290–296. 17662764 10.1016/j.jtcvs.2007.02.041

[87] Rosenthal NHR. Heart Development and Regeneration. Amsterdam: Acad Press; 2010.

[88] Yeh ML, Gonda Y, Mommersteeg MTM, Barber M, Ypsilanti AR, Hanashima C, Parnavelas JG, Andrews WD. Robo1 modulates proliferation and neurogenesis in the developing neocortex. J Neurosci 2014; 34:5717–5731. 24741061 10.1523/JNEUROSCI.4256-13.2014 PMC3988420

[89] Borrell V, Cardenas A, Ciceri G, Galceran J, Flames N, Pla R, Nobrega-Pereira S, Garcia-Frigola C, Peregrin S, Zhao Z, Ma L, Tessier-Lavigne M, Marin O, Cárdenas A, Ciceri G, Galcerán J, Flames N, Pla R, Nóbrega-Pereira S, García-Frigola C, Peregrín S, Zhao Z, Ma L, Tessier-Lavigne M, Marín O, Cardenas A, Ciceri G, Galceran J, Flames N, Pla R. Slit/Robo signaling modulates the proliferation of central nervous system progenitors. Neuron 2012; 76:338–352. 23083737 10.1016/j.neuron.2012.08.003 PMC4443924

[90] Liu J, Zhang L, Wang D, Shen H, Jiang M, Mei P, Hayden PS, Sedor JR, Hu H. Congenital diaphragmatic hernia, kidney agenesis and cardiac defects associated with Slit3-deficiency in mice. Mech Dev 2003; 120:1059–1070. 14550534 10.1016/s0925-4773(03)00161-8

[91] Long H, Sabatier C, Ma L, Plump A, Yuan W, Ornitz DM, Tamada A, Murakami F, Goodman CS, Tessier-Lavigne M. Conserved roles for Slit and Robo proteins in midline commissural axon guidance. Neuron 2004; 42:213–223. 15091338 10.1016/s0896-6273(04)00179-5

[92] Rhee J, Buchan T, Zukerberg L, Lilien J, Balsamo J. Cables links Robo-bound Abl kinase to N-cadherin-bound beta-catenin to mediate Slit-induced modulation of adhesion and transcription. Nat Cell Biol 2007; 9:883–892. 17618275 10.1038/ncb1614

